# Supplementary material for: Parameters for Optoperforation-Induced Killing of Cancer Cells Using Gold Nanoparticles Functionalized With the C-terminal Fragment of Clostridium Perfringens Enterotoxin
Source: Int J Mol Sci. 2019 Aug 30;20(17):4248. doi: 10.3390/ijms20174248 (PMC6747448; doi:10.3390/ijms20174248)
Supplement: Supplementary file 1 [file ijms-20-04248-s001.pdf]

Supplementary Materials

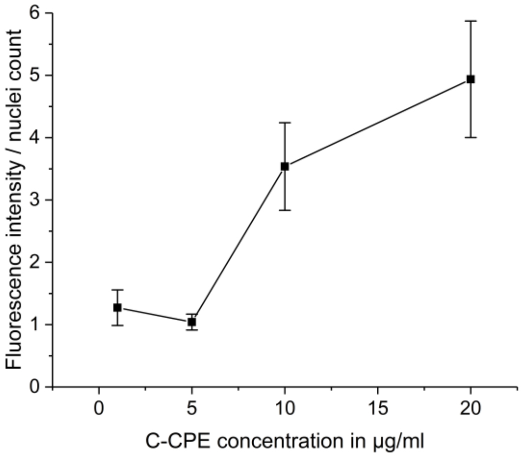

Figure 1. Binding of C-CPE to Caco-2 cells. For the binding analysis 1, 5, 10 and 20 µg/mL C-CPE were mixed with 30 µg/mL Strep-Tactin Chromeo 546 to generate Strep-Tactin Chromeo-C-CPE complex. Cells were incubated with the complex for 3 h. As control cells were incubated only with the Strep-Tactin Chromeo 546. Fluorescence intensity of the complexes treated cells and control cells was measured. Florescence intensity of the control cells was taken as background. For nuclei count, nuclei were stained with Hoechst. Graph represent the mean ± SEM of C-CPE-Strep-Tactin Chromeo 546 fluorescence normalized to the number of nuclei.

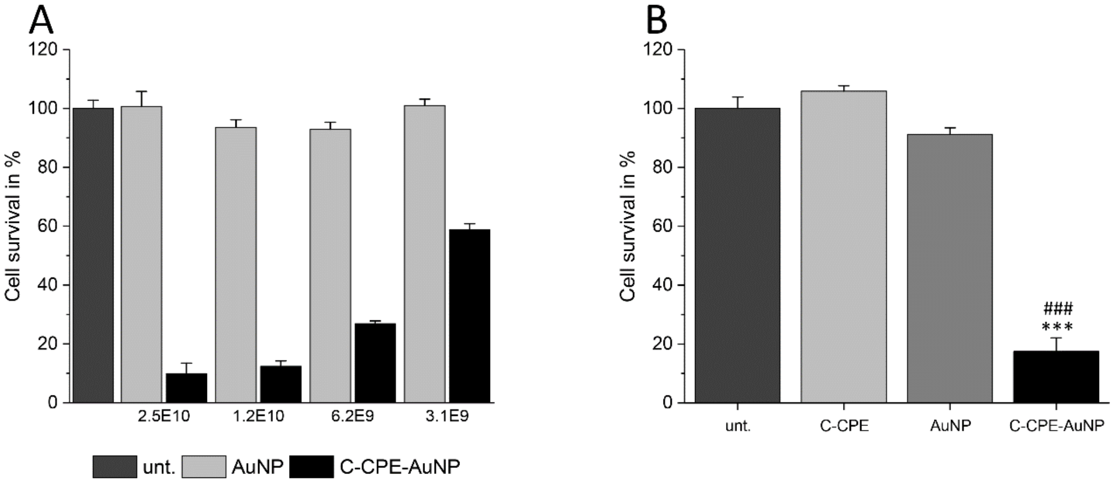

Figure 2. GNOME-LP efficiency depends on AuNP concentration and laser fluence. (**A**) Different AuNP concentrations as indicated were functionalized with 20 µg C-CPE and applied to Caco-2 cells before GNOME-LP (30 mW at 5 mm/s) application. GNOME-LP with an AuNP concentration lower than 1.2 × 10^{10} particle per ml caused more cell survival. (**B**) Caco-2 cells incubated with 2.5 × 10^{10} AuNPs per ml functionalized with 20 µg/mL C-CPE were irradiated three times with a laser fluence of 30 mW at 40 mm/s. The repetition of GNOME-LP application reduced the cell survival to similar extent than a laser fluence at 30 mW at 5 mm/s. the results were analyzed with Student's t test. * Significant difference to the untreated (unt.) control group: ***$p < 0.001$. # Significant difference to the AuNP control group: ### $p < 0.001$. All graphs represent the mean ± SEM of cell survival normalized to untreated cells.

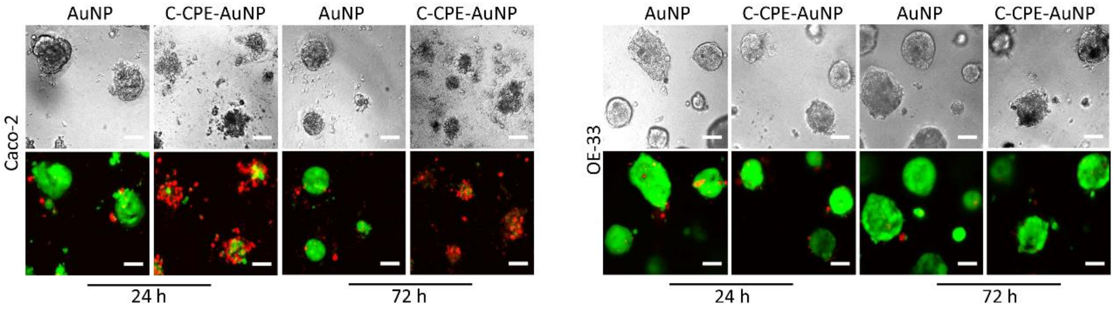

Figure 3. Confocal images of Caco-2 and OE-33 spheroids treated with GNOME-LP in presence of AuNP or C-CPE-AuNP after 24 h and 72 h treatment. Long-term effect of the C-CPE-AuNPs on Caco-2 spheroids showed increasing loss of spheroid structure and further reduced cell survival after treatment. For OE-33 spheroids the C-CPE-AuNPs had no long-term effect on spheroid formation after laser irradiations. Living cells are indicated by calcein AM (green) and death cells by propidium iodide (PI) uptake (red). Scale bar: 50 µm.

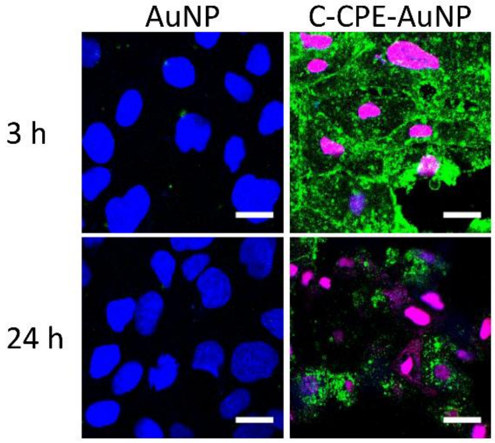

Figure 4. Annexin V Atto488 (green) and propidium iodide (red) staining of Caco-2 cells 3 h and 24 h after GNOME-LP (60 mJ/cm²; 5 mm/s). Nuclei stained with Hoechst (blue). Scale bar: 20 µm.

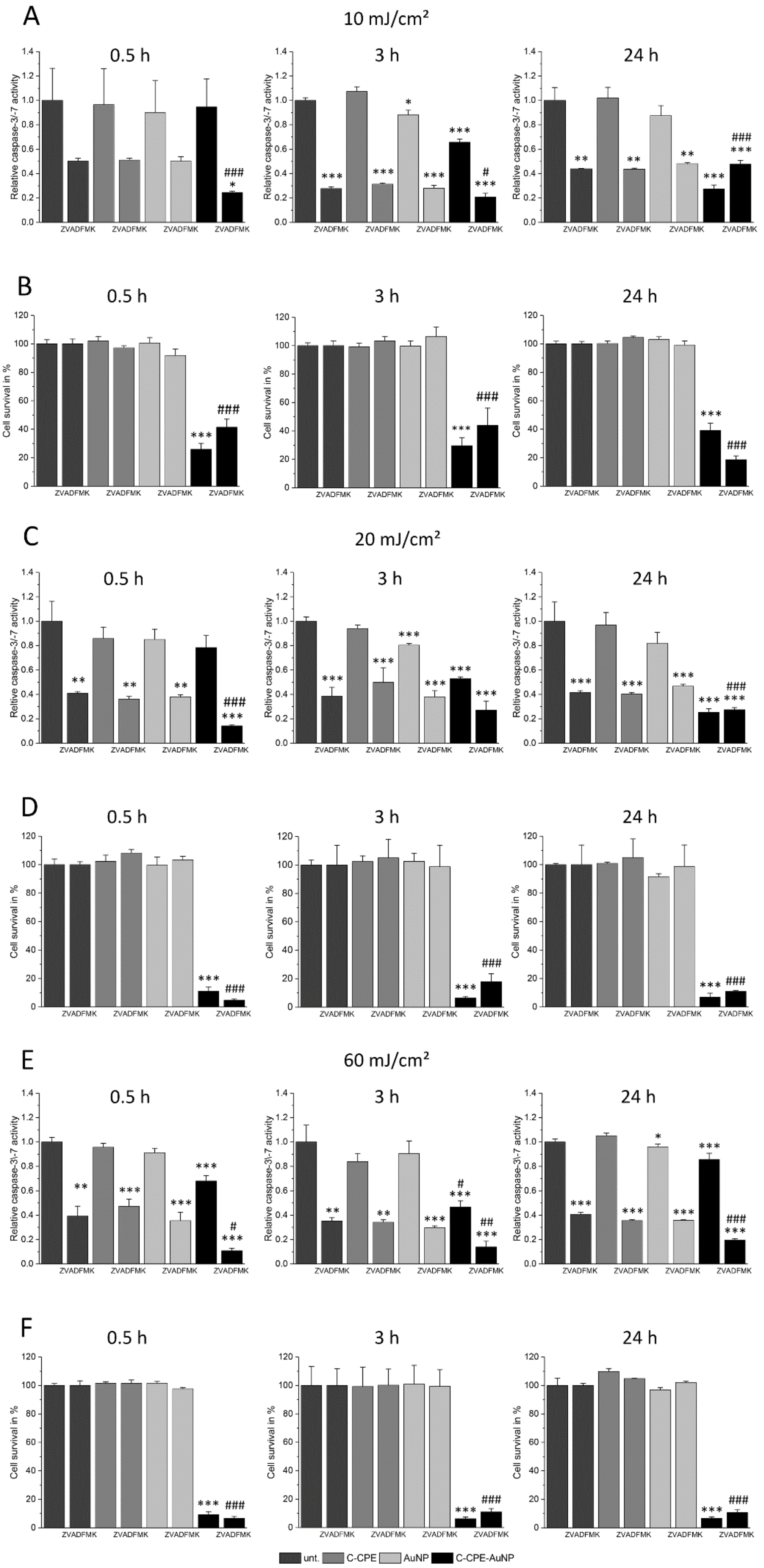
A
10 mJ/cm^2
0.5 h
3 h
24 h
Relative caspase-3/-7 activity
ZVADFMK
B
Cell survival in %
C
20 mJ/cm^2
D
E
60 mJ/cm^2
F
unt.
C-CPE
AuNP
C-CPE-AuNP

Figure 5. Activity of caspase 3/ 7 in Caco-2 cells after GNOME-LP treatment in presence of C-CPE-AuNPs. (**A**) and (**B**) GNOME-LP was applied at 10 mJ/cm^2 and 5 mm/s. (**C**) and (**D**) GNOME-LP was applied at 20 mJ/cm^2 and 5 mm/s. (**E**) and (**F**) GNOME-LP was applied at 60 mJ/cm^2 and 5 mm/s (**A**), (**C**), and (**E**) Caspase-3/-7 activity after GNOME-LP with or without the caspase inhibitor Z-VAD-FMK (20 μM). The columns represent the mean ± SEM of caspase-3/-7 activity relative to untreated cells without Z-VAD-FMK as control. The results were analyzed by Student's *t* test. * Significant difference to the control: *$p < 0.05$, **$p < 0.01$, ***$p < 0.001$. # Significant difference to the control reference with Z-VAD-FMK: #$p < 0.05$, ###$p < 0.001$. (**B**), (**C**), and (**F**) Cell survival after GNOME-LP with or without the caspase inhibitor Z-VAD-FMK. The columns represent the mean ± SEM of cell survival relative to untreated cells. The results analyzed by Student's t test. * Significant difference to untreated cells: ***$p < 0.001$. # Significant difference to untreated cells with Z-VAD-FMK: ###$p < 0.05$.
